# Establishment of an *Agrobacterium*‐mediated transformation system for the genetic engineering of *Linum grandiflorum* Desf.

**DOI:** 10.1111/ppl.70059

**Published:** 2025-01-20

**Authors:** Karol Gad, Hanna Levchuk, Christian Kappel, Michael Lenhard

**Affiliations:** ^1^ Institute of Biochemistry and Biology University of Potsdam Potsdam‐Golm Germany

## Abstract

Genetic transformation is a powerful tool in plant biotechnology. However, its application is limited to species that are well‐studied and easy to transform. There is a critical need to establish transformation protocols for non‐model species. A stable transformation method using *Agrobacterium rhizogenes* for hairy root transformation and regeneration of transgenic *Linum grandiflorum* was established. This protocol shows the successful co‐transformation of different T‐DNA fragments from both the native Ri plasmid and the binary vector with the reporter gene. Hairy roots were produced after inoculation with *Agrobacterium rhizogenes* from which later shoots were formed from the callus, and subsequently, whole plants were regenerated. This protocol significantly facilitates genomic studies in *Linum grandiflorum*, particularly in investigating genes at the *S*‐locus supergene, which are crucial for understanding self‐incompatibility. Moreover, the established transformation method enables the production of hairy root lines, which can be utilized for the biosynthesis of medically useful and commercially valuable plant metabolites.

## 1 INTRODUCTION

*Linum grandiflorum* is an annual species of flax known for its red flowers that show style length dimorphism. This interesting floral polymorphism was already mentioned and described by Darwin in 1863 in his work on heterostyly in the genus *Linum* (Darwin, 1863). In *L*. *grandiflorum* populations, short‐ and long‐styled morphs co‐occur. This floral characteristic is linked to the *S*‐locus supergene, which also controls self‐incompatibility. *L. grandiflorum* is a prime model for studying stigma‐height dimorphism and has been used for many years to investigate its molecular and genetic basis (Ghosh & Shivanna, 1980; Lewis, 1943; Murray, 1986, Ushijima et al., 2012).

Moreover, *L. grandiflorum* is an interesting scientific object due to its secondary metabolites, such as lignans, neolignans, and flavonoids, with potential anti‐inflammatory and antioxidant properties (Mohammed et al., 2009; Schmidt et al., 2010, 2012). *L. grandiflorum* seeds are rich in fatty acids, mainly α‐linolenic acid, as well as less abundant linoleic acid and oleic acid (Mohammed et al., 2018; Rogers, 1972). Triacylglycerols contained in its oil have promising results for their cytotoxicity and hepatoprotective activity (Mohammed et al., 2018).

Plant biotechnology is a field of science that aims to improve plants via genetic manipulation by modulating, deleting existing or inserting new genes. Over many years, many different methods and techniques have been used to change gene expression in various plants. In general, each species to be transformed requires the development of a customized protocol, and for most species, this is associated with the establishment of plant tissue culture.

The most commonly used method is *Agrobacterium*‐mediated transformation, which relies on the natural ability of this bacterium to introduce part of its DNA into a plant genome. *A. tumefaciens* is the best characterized and widely used species in laboratory practice to generate genetically modified plants. The closely related *A. rhizogenes* provides an equally powerful yet less popular tool. This species can introduce a part of its root‐inducing (Ri) plasmid DNA, known as transferred DNA (T‐DNA), to cause hairy root disease (Chilton et al., 1982). Depending on the strain, the Ri plasmid has one T‐DNA, or two different fragments named TL‐DNA, containing *rolA*, *rolB*, *rolC*, *rolD* genes, and TR‐DNA, carrying *aux1*, *aux2* and opine biosynthesis genes (Camilleri & Jouanin, 1991; Spena et al., 1987; Vilaine & Casse‐Delbart, 1987). The *rolB* gene encoding a plasma membrane protein is essential for the formation of transformed hairy roots in infected plants (Spena et al., 1987). Its mechanism of action is poorly understood, but it is likely to increase the auxin sensitivity of cells, which leads to root formation without externally added plant hormones (Veena & Taylor, 2007). Plants regenerated from transformed hairy roots exhibit more compact growth habits, which are beneficial for breeding programs of many ornamental and crop species (Rüter et al., 2024; Desmet et al., 2024; Ricci et al., 2023). The use of *A. rhizogenes* relies on the possibility of further introduction of commonly used binary vectors and efficient transformation (Supplementary Figure S1) (Liu et al., 2024; Ramasamy et al., 2023; Boisson‐Dernier et al., 2001).

## 2 MATERIALS AND METHODS

### 2.1 Materials

#### 2.1.1 Plant material

*L. grandiflorum* seeds were obtained from plants growing in the Botanical Garden of the University of Potsdam (Maulbeerallee 2, 14469 Potsdam, Germany), which were raised from seeds originally harvested from their natural habitat many years ago, though they lack an accession number.

#### 2.1.2 Plant growth media

All culture media were autoclaved at 121°C under a pressure of 2 atm for 15 minutes and stored at room temperature. Prior to use, the media were liquefied using a microwave. Plant hormones and heat‐sensitive chemicals were added after autoclaving. To remove *Agrobacterium*, the media used after transformation were supplemented with 250 mg L^‐1^ timentin (ticarcillin disodium/potassium clavulanate) and 0.1% PPM.

MS20: 4.4 g L^‐1^ Murashige & Skoog medium including Gamborg B5 vitamins, 2% (w/v) sucrose, 8 g L^‐1^ agar, pH 5.8

MS30: 4.4 g L^‐1^ Murashige & Skoog medium including Gamborg B5 vitamins, 3% (w/v) sucrose, 8 g L^‐1^ agar, pH 5.8

SIM (shoot induction medium): 4.4 g L^‐1^ Murashige & Skoog medium including Gamborg B5 vitamins, 3% (w/v) sucrose, 1 mg L^‐1^ BAP, 20 mg L^‐1^ adenine hemisulfate, 8 g L^‐1^ agar, pH 5.8

Root induction medium: 4.4 g L^‐1^ Murashige & Skoog medium including Gamborg B5 vitamins, 2% (w/v) sucrose, 0.1 mg L^‐1^ IBA, 8 g L^‐1^ agar, pH 5.8

Co‐cultivation medium: 4.4 g L^‐1^ Murashige & Skoog medium including Gamborg B5 vitamins, 3% (w/v) sucrose, 200 μM acetosiringone, 8 g L^‐1^ agar, pH 5.8

#### 2.1.3 Bacterial strain

*Agrobacterium rhizogenes* A4 (CECT 478) was obtained from the Spanish Type Culture Collection (CECT) at the University of Valencia. This strain is an agropine‐type, possessing the root‐inducing plasmid (pRiA4).

#### 2.1.4 Bacterial growth media and conditions

The bacterial cultures were grown in TY medium, which consists of 5 g L^‐1^ tryptone, 3 g L^‐1^ yeast extract, 0.9 g L^‐1^ CaCl_2_ × 2H_2_O. The pH of the medium was adjusted to 7.2. The solid medium contained 15 g L^‐1^ agar. The bacterial cultures were incubated at 26°C with continuous shaking at 220 rpm.

#### 2.1.5 Plasmid

The 35S:RUBY plasmid was obtained from Addgene and was a gift from Yunde Zhao (Addgene plasmid # 160908). The T‐DNA encodes the reporter *RUBY* under the control of the Cauliflower Mosaic Virus (CaMV) 35S promoter. *RUBY* converts tyrosine to vividly red betalain. Additionally, the plasmid includes the *hygromycin phosphotransferase* gene for selection.

### 2.2 Flax transformation

#### 2.2.1 Establishment of plant tissue culture

The seeds were surface sterilized with 70% ethanol for 2 minutes, followed by 25% PPM solution for 30 minutes. Subsequently, the seeds were rinsed with sterile water and sown on plates with MS20 before being transferred to the growth chamber. The plants were grown under long‐day conditions (16/8 h light/dark) at 20°C.

#### 2.2.2 Bacteria preparation

The integrity of the 35S:RUBY plasmid was checked by digestion with *Hind*III, *Spe*I, *Bsr*GI enzymes and Sanger sequencing. The plasmid was transformed into an electrocompetent *Agrobacterium* strain using the Micropulser Electroporator (Bio‐Rad). Bacteria were streaked on a plate with TY medium supplemented with 50 mg L^‐1^ spectinomycin. A single colony was inoculated in 5 mL of TY supplemented with antibiotics and incubated with shaking at 26°C for two days. A bacterial stock was prepared from 1 mL of saturated culture mixed with 70 μL of DMSO, which was frozen and stored at ‐80°C.

#### 2.2.3 Hairy root transformation

*A. rhizogenes* A4 from the stock was streaked on a plate with TY medium supplemented with 50 mg L^‐1^ of spectinomycin. A single colony was inoculated in 5 mL of TY with antibiotics and incubated with shaking at 26°C for two days. One day before transformation, the culture was refreshed by transferring 100 μL of saturated culture to 100 mL of fresh medium and incubating overnight at 220 rpm at 26°C to reach an OD_600_ of 0.8. Afterwards, the culture was centrifuged for 15 minutes at 5,403 *g*. The supernatant was carefully discarded, and the bacteria were washed twice with a liquid co‐cultivation medium and resuspended in 100 mL. The OD was measured on the BioPhotometer (Eppendorf) and adjusted to 0.1. The suspension was incubated without shaking in the dark for 2.5 h prior to transformation. Ten‐day‐old flax seedlings were used for transformation. The hypocotyls and cotyledons were cut into small segments that were immersed and incubated in a bacterial suspension for 10 minutes with gentle shaking. Later, the explants were dried and placed on the co‐cultivation medium for 2 days in the dark. To remove excess *Agrobacterium*, explants were washed in sterile water supplemented with 350 mg L^‐1^ of timentin, 0.1% PPM for 15 minutes and placed on MS30 until transgenic roots were formed. The medium was changed every 2 weeks.

#### 2.2.4 Shoot regeneration

Transgenic roots characterized by vivid red colouration were cut into small segments and transferred onto SIM. The medium was changed every 2 weeks, until the shoots started to develop.

#### 2.2.5 Rooting and acclimatization

To stimulate root formation, the shoots were stripped of their lower leaves and transferred to sterile glass jars with a root induction medium. Afterwards, well‐rooted plants were transferred into soil and covered with a plastic lid for 6 days to acclimatize them to greenhouse conditions. The plants were grown under long‐day conditions with additional artificial light (16/8 h light/dark) 22‐26°C during the day and 18‐20°C at night.

### 2.3 Molecular analysis of transformants

#### 2.3.1 DNA isolation

Genomic DNA was isolated from leaves using the CTAB method. Leaves were harvested into Eppendorf tubes and immediately placed in liquid nitrogen. The samples were ground using a mortar and pestles. The tissue powder was mixed with 750 μL of extraction buffer (100 mM Tris pH 8.0, 2% (w/v) CTAB, 30 mM EDTA, 2 M NaCl, 2% PVP) and incubated with shaking for 30 minutes at 65°C. Then, samples were mixed with 750 μL of chloroform:isoamyl alcohol (24:1), shaken for 5 minutes and centrifuged at 17,949 *g* for 5 minutes at 4°C. Then, 500 μL of aqueous phase was collected into new Eppendorf tubes and mixed with 500 μL of isopropanol and 50 μL of 3 M NaOAc (pH 5.2). The samples were vortexed, incubated for 5 minutes at room temperature and centrifuged at 17,949 *g* for 10 minutes. The supernatant was discarded, and the pellet was washed with 1 mL of 70% ethanol. The pellet was dried and dissolved in 100 μL of TE with RNase.

#### 2.3.2 PCR

PCR was carried out using MyTaq™ DNA Polymerase (Meridian Bioscience) in the thermocycler (Eppendorf). The reaction set‐up and conditions were performed according to the manufacturer's manual. Oligonucleotide sequences are listed in the Supplementary File S1. The PCR products were analyzed by electrophoresis on a 1% agarose gel containing ethidium bromide and visualized under UV light.

#### 2.3.3 RNA isolation and sequencing

Leaves were harvested into Eppendorf tubes and immediately placed in liquid nitrogen. Samples were ground using a mortar and pestle. RNA was isolated using the RNeasy Plant Mini Kit according to the manufacturer's manual. RNA concentration and quality, as assessed by 260/230 and 260/230 ratios, were determined using the DeNovix DS‐11 spectrophotometer. Afterwards, samples were sent for RNA sequencing.

RNA‐seq reads were mapped against the theoretical transcript sequences inserted in the 35S:RUBY and pRiA4 constructs using BWA‐MEM (Li, 2013). Specifically, for 35S:RUBY, the targeted transcripts included *CYP76AD1*, *DODA*, *Glucosyltransferase*, and *hpt*. For pRiA4, the targeted transcripts included X12579.1, OQ673858.1, MT514512.1, X64255.1:5795‐6391, *aux1*, and *aux2*. The mappings were sorted, indexed, and quantified using the Samtools (Li et al., 2009) sub‐programs: sort, index, and idxstats, respectively. Counts were normalized relative to the total number of reads. All analyses and figure generation were performed in R (REF).

#### 2.3.4 Whole genome sequencing

Genomic DNA was isolated from young shoots using the CTAB method. The quality of the isolated DNA was assessed by measuring the 260/230, and 260/230 absorbance ratios using the DeNovix DS‐11 spectrophotometer to confirm sample purity. DNA concentration was quantified using Invitrogen™ Qubit™ dsDNA Assay‐Kits. Afterwards, samples were sent for DNA sequencing.

DNA‐seq reads were mapped against a reference combining the *Linum usitatissimum* genome (GCA_030674075.2) and the full 35S:RUBY and pRiA4 constructs using BWA‐MEM. The mappings were sorted and indexed using Samtools and then visualized in IGV (Thorvaldsdóttir et al., 2013). Transitions to the genome were identified by examining reads that were only partially mapped against the 35S:RUBY and pRiA4 constructs in IGV. One transition sequence was reconstructed by assembling individual reads based on the perfect overlap at the ends, identifying a transition from the construct to the genome sequence. This transition was confirmed using PCR and Sanger sequencing. Oligonucleotide sequences are listed in the Supplementary File S1.

#### 2.3.5 Betalain extraction

To determine the betalain concentration, shoots were harvested (40 mg) and ground in liquid nitrogen using the MM301 Retsch Mixer Mill. The betalain was extracted using 40% methanol containing 50 mM sodium ascorbate and purified as previously described by Liu et al., 2024 for the quantitative *RUBY* reporter assay. The absorbance was measured using the DeNovix DS‐11 spectrophotometer.

## 3 RESULTS

*A. rhizogenes*‐mediated transformation consists of two steps, hairy root transformation and whole plant regeneration from root‐derived explants. In our study, *L. grandiflorum* was transformed with *A. rhizogenes* A4, an agropine‐type strain carrying the native pRiA4 plasmid. This strain was made electrocompetent and transformed with the 35S:RUBY binary vector that encodes *CYP76AD* (*P450 oxygenase CYP76AD*), *DODA* (*L‐DOPA 4,5‐dioxygenase*), and *GT* (*glucosyltransferase*) genes for the biosynthesis of the vivid red betalain. This enables a straightforward differentiation between transformed and non‐transformed plant cells (He et al., 2020).

**FIGURE 1:**
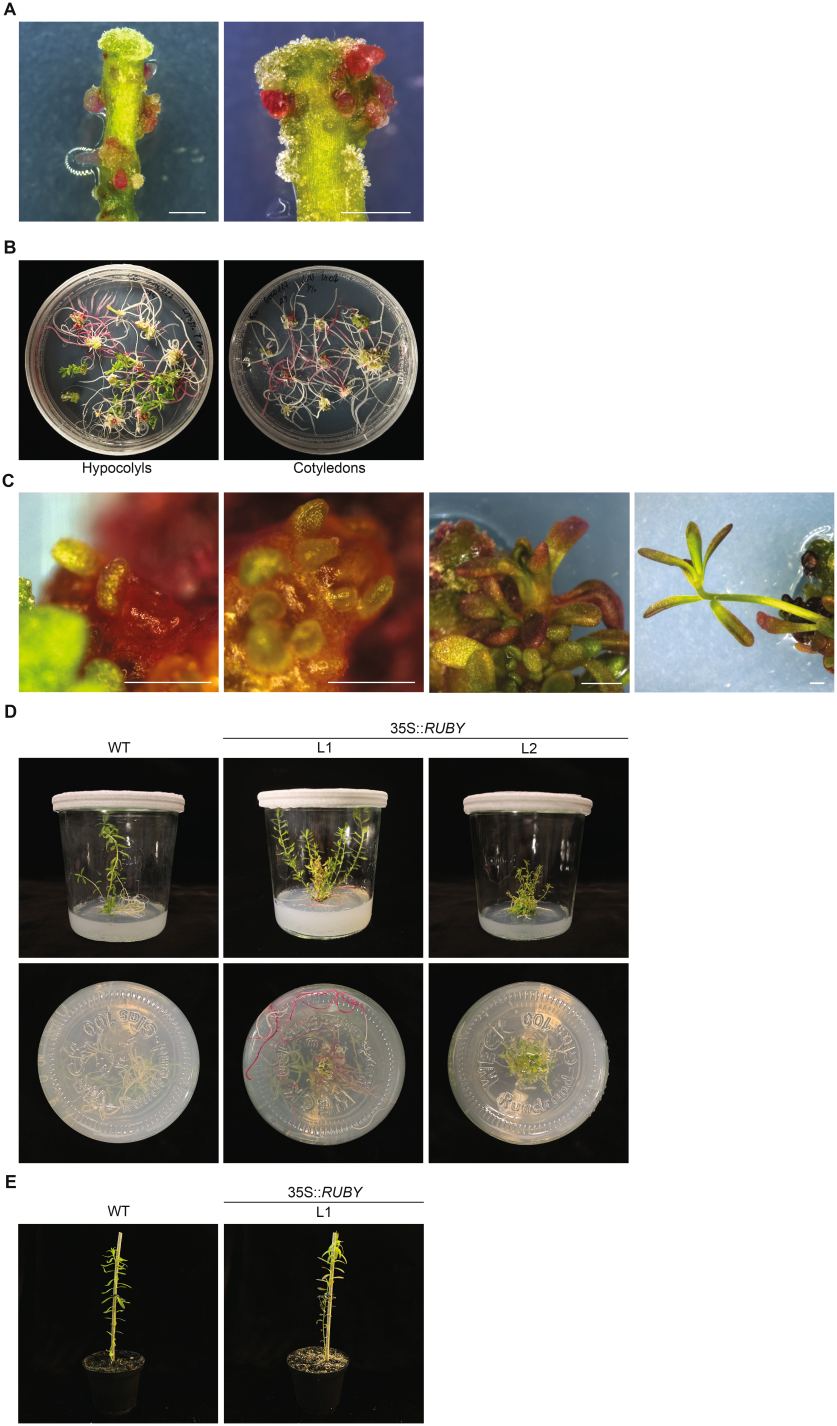
Hairy root transformation and transgenic shoots regeneration of *L. grandiflorum*. (A) Hairy root formation on hypocotyl explants after inoculation with *A. rhizogenes*. Both *RUBY* expressing (red) and non‐expressing roots (white) are visible. Scale bars are 1 mm. (B) *RUBY* expressing (red) and non‐expressing (white) hairy roots induced on hypocotyl and cotyledons‐derived explants. (C) Transgenic shoot formation on the shoot‐inducing medium. Scale bars are 1 mm. (D) Regeneration of transgenic plants *in vitro*. WT: wild‐type *L. grandiflorum*, L1, 2: transgenic line 1, 2. (E) Acclimatized plants after 4 weeks from their transfer to the greenhouse. WT: wild‐type *L. grandiflorum*, L1: transgenic line 1.

Hairy roots were produced approximately 2 weeks after inoculation with *A. rhizogenes* (Figure 1A). White roots are induced by the expression of the *rolB* gene, which is present in the TL‐DNA of the pRiA4 plasmid, while red roots are formed due to the expression of genes from the TL‐DNA fragment of pRiA4 and the T‐DNA from the 35S:RUBY plasmid.

To test the ability of hairy root induction on different explants, we transformed 40 hypocotyl and 35 cotyledon explants with *A. rhizogenes* carrying the pRiA4 and the 35S:RUBY plasmids. Of the hypocotyl explants, 32 (80%) formed a total of 264 hairy roots; of these, 165 remained white, and 99 (38%) turned red (Table 1A). Of the cotyledon explants, 22 (63%) formed roots. A somewhat lower percentage of these cotyledon‐derived roots were red (51/179; 28%) compared to the hypocotyl‐derived ones. However, in our experiment, we did not test the shoot regeneration ability of cotyledon‐derived explants. During hairy root induction, the formation of non‐transformed shoots was observed on hypocotyls, while cotyledons were not prone to regenerate any (Figure 1B).

**TABLE 1A:** Efficiency of transformed hairy root formation.

Explant type	Number of tested explants	Number of explants forming hairy roots	Numbers of white roots	Number of red roots
Hypocotyls	40	32	165	99
Cotyledons	35	22	128	51

**FIGURE 2:**
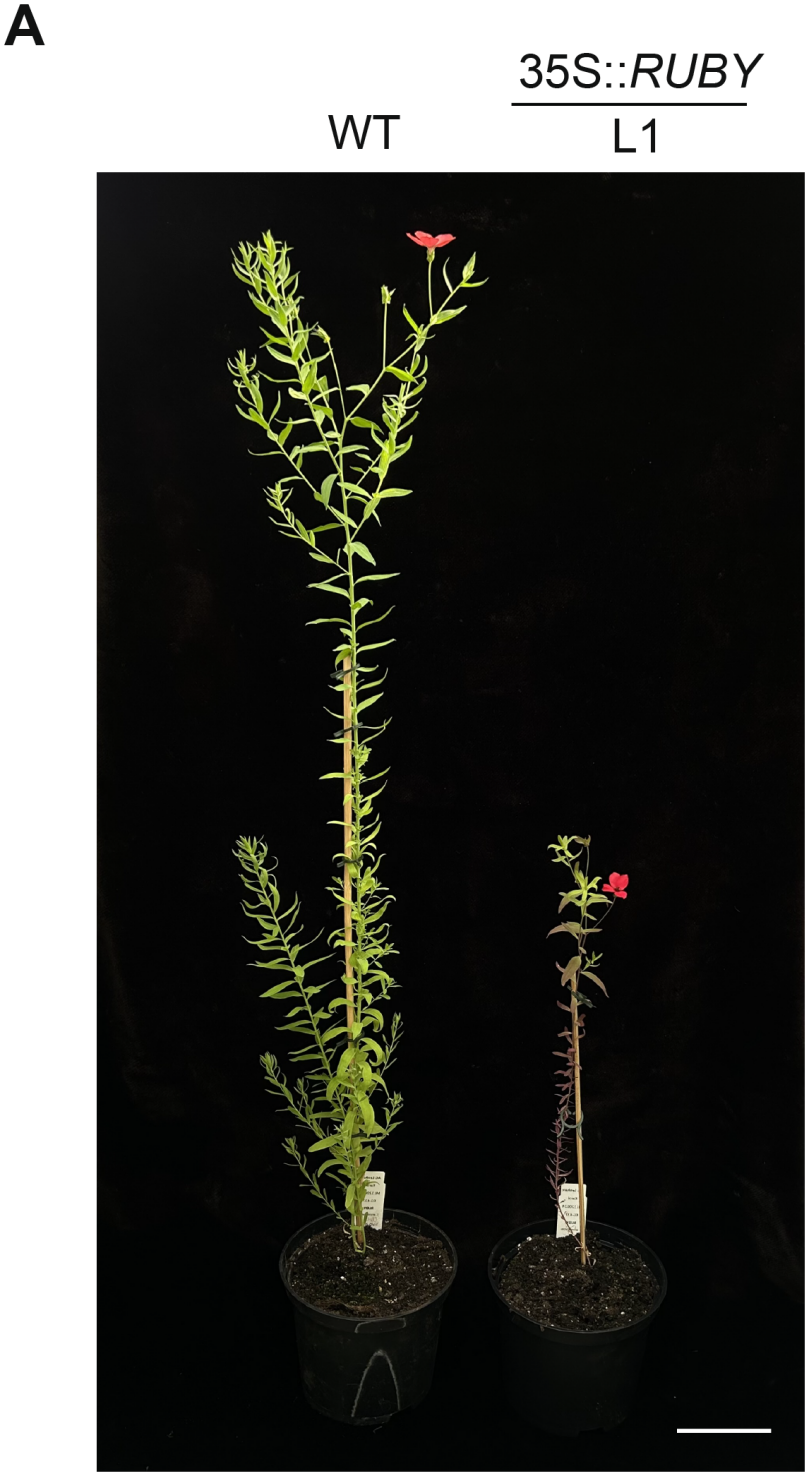
Flowering of *L. grandiflorum*. (A) Flowering of *L. grandiflorum* 8 weeks after transfer to greenhouse conditions. WT: wild‐type *L. grandiflorum*, L1: transgenic line 1. The scale bar is 10 cm.

To test shoot formation, we generated 490 segments from red hypocotyl‐derived hairy roots and placed them on SIM. Of these, 5 root segments (1%) formed transgenic shoots (Table 1B). Shoots were formed on SIM after approximately 4‐6 weeks and subsequently were cut from explants and moved onto MS20 to allow their elongation (Figure 1C). We observed the successful regeneration of shoots from five independent transgenic roots. Shoots were produced from the callus of root‐derived explants that originated from separate transformation events. Among these, two plants named L1 and L2 exhibited robust growth and were propagated *in vitro* as promising candidates for acclimatization to greenhouse conditions. L1 was characterized by normal morphology and red colouration of leaves and vividly red roots, whereas L2 displayed a dwarf phenotype with short internodes, increased branching, green colour with only slight tinges of red, curled leaves and white roots (Figure 1D). Line L1, with its presumed strong expression of 35S::*RUBY* was selected for further experiments. Roots were formed on the root‐inducing medium after approximately 4‐6 weeks. Subsequently, the plants were successfully acclimatized to greenhouse conditions, transplanted into the soil (Figure 1E), and began producing flowers after 8 weeks (Figure 2A). Plants regenerated *in vitro* following transformation showed slower growth and smaller stature compared to wild‐type (WT) plants raised from seeds, which we ascribe to the effect of stress during *in vitro* regeneration. Ultimately, transformants also grew to a wild‐type height (Figure S2A, B).

**TABLE 1B:** Efficiency of transgenic shoot formation.

Explant type	Number of roots segments placed on SIM	Number of roots forming buds
Hypocotyls	490	5

**FIGURE 3:**
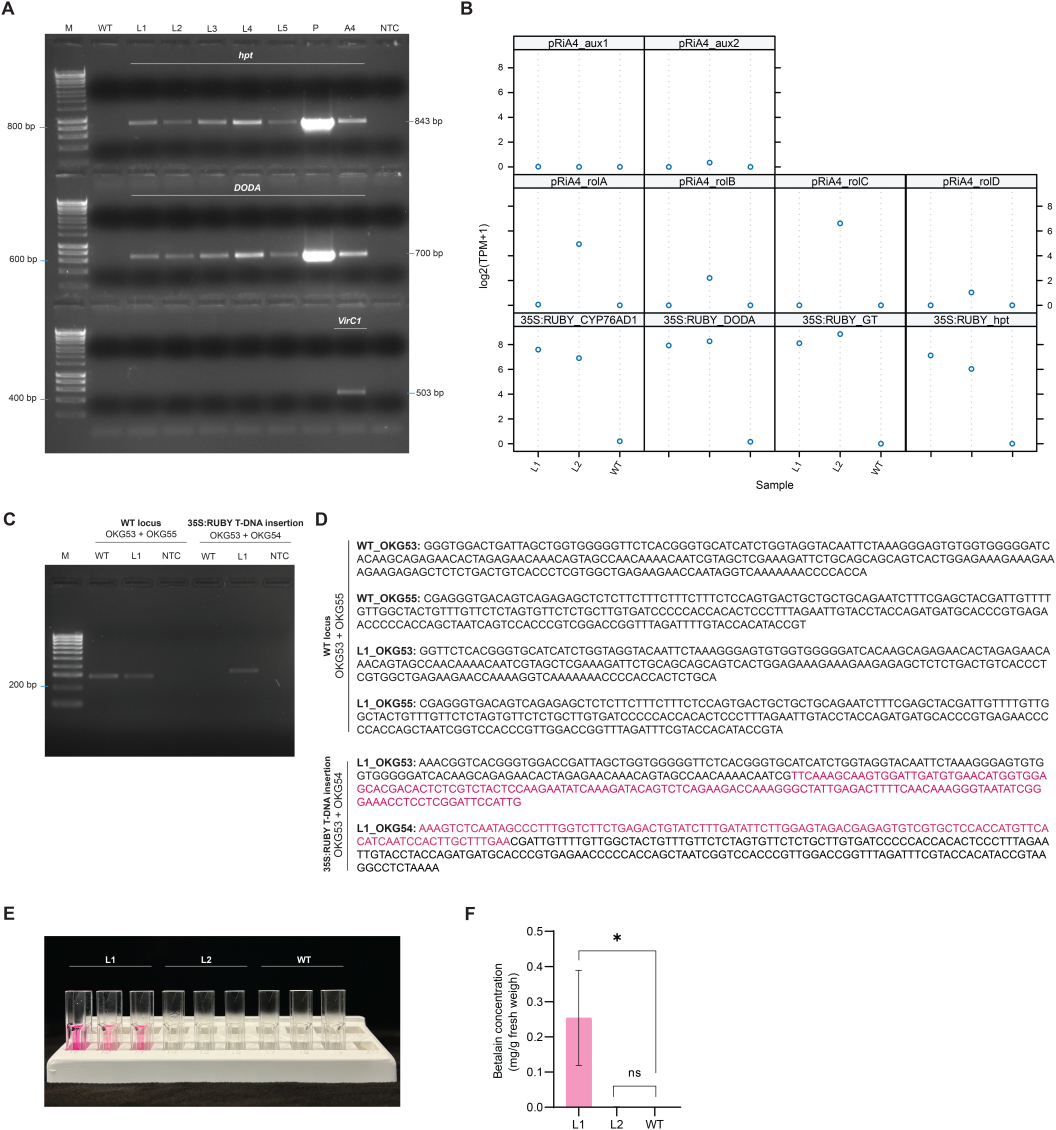
Molecular analysis of transgenic *L. grandiflorum*. (A) The presence of introduced transgenes was detected by PCR using primers against indicated targets: *hpt* (*hygromycin phosphotransferase*), *DODA* (*L‐DOPA 4,5‐dioxygenase*), *VirC1*. M: molecular marker, WT: wild‐type *L. grandiflorum*, samples L1‐L5: five independent transgenic lines, sample P: 35S:RUBY plasmid as a positive control, A4: *A. rhizgenes* used for transformation as a negative control for contamination with bacteria, NTC: no template control. (B) Transgene expression was analysed using RNA‐seq data. Expression level of *rolA*, *rolB*, *rolC*, *rolD, aux1*, and *aux2* derived from TL‐DNA and TR‐DNA of the pRiA4 plasmid, while *CYP76AD* (*P450 oxygenase CYP76AD*), *DODA* (*L‐DOPA 4,5‐dioxygenase*), *GT* (*glucosyltransferase*), *hpt* (*hygromycin phosphotransferase*) are derived from the T‐DNA of the 35S:RUBY plasmid. L1, L2: two independent transgenic lines, WT: wild‐type *L. grandiflorum*. (C) Confirmation of the T‐DNA insertion site using PCR against WT allele and T‐DNA insertion allele. M: molecular marker, WT: wild‐type *L. grandiflorum*, L1: transgenic line 1, NTC: no template control. (D) Confirmation of the T‐DNA insertion using Sanger sequencing. WT: wild‐type *L. grandiflorum*, L1: transgenic line 1. DNA sequences of the T‐DNA fragments derived from 35S:RUBY were marked in magenta. (E) Betalain extracts after extraction from shoots. L1, L2: two independent transgenic lines, WT: wild‐type *L. grandiflorum*. (F) Quantitive measurement of the betalain concentration. L1, L2: two independent transgenic lines, WT: wild‐type *L. grandiflorum*. L1, L2: two independent transgenic lines, WT: wild‐type L. grandiflorum. The asterisk indicates a significant difference from WT by Student's t‐test with *P < 0.05, whereas nsP > 0.05. n=3.

To confirm the presence of transgenes, regenerated shoots were checked by PCR, testing for the *hpt* (*hygromycin phosphotransferase*) and *DODA* genes (Figure 3A). As the control for *Agrobacterium* contamination, the *VirC1* gene was checked. The presence of specific bands of the expected size for *hpt* and *DODA* confirmed the integration of the T‐DNA derived from the 35S:RUBY plasmid. DNA from *A. rhizogenes* A4 culture utilized for transformation was used as the contamination control. No contamination in the transgenic shoots was detected by PCR.

To investigate the expression profile of genes introduced via *Agrobacterium*‐mediated transformation, both L1 and L2 transgenic plants were analyzed by RNA‐seq. The gene expression of *CYP76AD*, *DODA*, *GT* and *hpt* was detected in both transgenic plants (Figure 3B). In L2, genes of TR‐DNA derived from pRiA4, such as *aux2*, showed only minimal expression, while the *aux1* transcript was not detected. Genes derived from the TL‐DNA of pRiA4, such as *rolA*, *rolB*, *rolC*, and *rolD* were strongly expressed in L2 and absent in L1, which is consistent with the strong morphological phenotype of the former but not the latter. The expression of transgenes introduced as three different T‐DNA fragments derived from the pRiA4 and 35S:RUBY plasmids confirmed the successful transformation of *L. grandiflorum*.

Whole genome sequencing was performed to validate the T‐DNA insertion. Mapping the reads against the 35S:RUBY plasmid indicated T‐DNA insertions in both lines. The RiA4 T‐DNA insertions were identified in line L2 (Figure S3A, B, and C). For line 1, we used the data to reconstruct one genomic locus where the 35S:RUBY fragment had integrated (Supplementary File S2). Specific primers were designed to amplify the flanking region in L1. The resulting PCR products were subjected to Sanger sequencing, which confirmed the T‐DNA integration sites (Figure 3C, D; Supplementary Data Set S1).

The concentration of betalain in the plants was determined via spectrophotometric analysis. L1 exhibited the highest absorbance signal, quantified as 0.254 mg of total betalain per g of fresh weight. In contrast, the wild‐type and L2 plants showed no betalain (Figure 3E, F). These results are consistent with the phenotype observed in the regenerated plants, where L1 plants exhibited intense red colouration.

**FIGURE 4:**
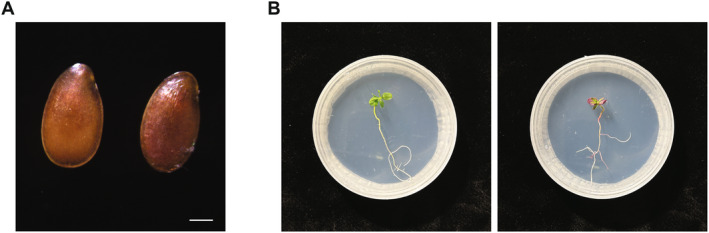
Stable transgene transmission to the next generation. (A) T1 seeds obtained from the cross between wild‐type *L. grandiflorum* and L1 transgenic line. Left is a presumed non‐transgenic seed, while right is a transgenic seed. Scale bar is 1 mm. (B) T1 seedlings germinated *in vitro*. Left is a non‐transgenic seedling, while right is a transgenic seedling.

To confirm the stable transgene transmission to the next generation, transgenic plants were crossed with wild‐type plants. Using L1 as a pollen donor, seed capsules were formed and harvested after approximately 5 weeks before dehiscence. Betalain accumulation was visible in T1 seeds (Figure 4A). Subsequently, the seeds were germinated *in vitro*. Among 36 seeds, 27 (75%) germinated, with 10 (37%) seedlings showing red colouration derived from expression of the *RUBY* reporter (Figure 4B). The observed segregation ratio of transgene inheritance in the progeny indicates a single heterozygous T‐DNA insertion.

Collectively, comprehensive analyses of the transgenic plants demonstrate the reliability of this protocol for *L. grandiflorum* transformation.

## 4 DISCUSSION

To the best of our knowledge, this is the first report of a generation of transgenic *L. grandiflorum*, including whole‐plant regeneration from transformed hairy roots induced by *A. rhizogenes*.

Hairy roots are highly promising explants because they are initiated from individual transformed cells and thus enable the generation of non‐chimeric plants (Bercetche et al., 1987). This system provides benefits over transformation with *A. tumefaciens*, for which the major problem is the formation of escapes. These non‐transformed shoots generate many plants that need to be screened for the presence of transgenes (Mlynárová et al., 1994). The regeneration of chimeric shoots makes this process even more difficult, as those plants are composed of transformed and untransformed cells. While they may have positive PCR results, they exhibit irregular segregation ratios in their progeny (Dong & McHughen, 1993).

Hairy root cultures provide tools for studying plant biology and also for plant biotechnology to produce secondary metabolites and recombinant proteins (Bourgaud et al., 2001). For several *Linum* species, such as *L. album* (Cong et al., 2015), *L. austriacum* (Mascheretti et al., 2021), *L. flavum* (Oostdam et al., 1993), *L. lewisii* (Dougué Kentsop et al., 2022), and *L. perenne* (Jullian‐Pawlicki et al., 2015) hairy root cultures that produce different secondary metabolites have been successfully established. Furthermore, it has been demonstrated that the natural transformation with the Ri plasmid and regeneration of *L. usitatissimum* are possible (Zhan et al., 1988).

Plants regenerated from hairy roots can exhibit altered phenotypes, including dwarfism or semi‐dwarfism, short internodes, curly leaves, prolific root formation and reduced apical dominance, because of the expression of hormone biosynthesis genes from the root‐inducing T‐DNA (Tepfer, 1984). This seems to be the case in our experiment for line 2, which expressed the *rol* hormone biosynthesis genes, correlating with their stunted growth. However, it is possible to regenerate plants with normal morphology that express only the desired transgene, but not the genes from the T‐DNA of the Ri plasmid. This seems to be the case for line 1.

In our transformation experiment, we observed that line L1 was shorter than the wild type. The transgenic plants, which were clonally propagated, exhibited reduced vigour and slower growth compared to wild‐type plants. This variation in growth can be influenced by changes in the transcriptome and metabolome caused by betalain synthesis. In transgenic carrot and tobacco, betanin synthesis notably affected photosynthesis and tyrosine metabolism, which are critical processes that can influence overall plant growth and development (Jiang et al., 2024; Wang et al., 2023).

The shoot regeneration efficiency in our experiment was comparable to the results obtained in *L. usitatissimum*, which varied from 0 to 6%. This efficiency depended on the flax cultivar and *A. rhizogenes* strain (Zhan et al., 1988). Earlier reports have assessed the regeneration capabilities of *L. grandiflorum* in tissue culture. It was shown that this species is more difficult to regenerate compared to other members of the genus *Linum*. The callus derived from protoplasts failed to form shoots, and rhizogenesis was more common (Barakat & Cocking, 1985). Despite the challenges of regenerating *L. grandiflorum in vitro*, we successfully induced indirect shoot organogenesis from root‐derived explants, followed by whole plant regeneration.

The overall efficiency of transgenic shoot formation was low (1%), especially when compared to the *Agrobacterium tumefaciens*‐mediated transformation method for cultivated flax, which typically gives an efficiency of several percent to 25% (Mlynárová et al., 1994). Therefore, further optimization of the regeneration medium composition, including the supplementation of different plant growth regulators or growth conditions, could enhance the shoot regeneration (Oguz 2024; Chen & Dribnenki, 2002; Mundhara & Rashid, 2002) Moreover, it was shown that peptides can enhance the regeneration and transformation efficiency of some recalcitrant plant species (Bennur et al., 2024; Yang et al., 2024).

## 5 CONCLUSION

In conclusion, we have established a successful method for *L. grandiflorum* transformation using *A. rhizogenes* and whole‐plant regeneration from transgenic roots. The establishment of a transformation system provides tools for investigating the genetic and molecular control of genes at the *S*‐locus supergene in *Linum*. Additionally, the described root transformation method enables the establishment of hairy root lines that can later be used for the production of plant metabolites and recombinant proteins.

## AUTHOR CONTRIBUTIONS

Conceptualization, K.G. and M.L.; investigation and methodology, K.G.; formal analysis and resources, K.G., H.L., C.K., M.L.; writing – original draft, K.G.; writing – review and editing, K.G. and M.L.

## FUNDING INFORMATION

This work was supported by the Deutsche Forschungsgemeinschaft (grant number 408296963).

## ETHICAL DECLARATIONS

Not applicable

## Supporting information


**Supplementary Data Set S1.** Sanger sequencing results

**Supplementary Figure S1.** **Schematic representation of *Agrobacterium rhizogenes* and the Ri plasmid**.
*Agrobacterium rhizogenes* strain A4 is an agropine type strain that contains a root‐inducing (Ri) plasmid with two distinct T‐DNA fragments: TL‐DNA, which is involved in the biosynthesis of auxins, and TR‐DNA, which carries opine biosynthesis genes. Commonly used binary vectors, such as 35S:RUBY, are designed to carry foreign genes of interest and can be additionally introduced into the host plant genome. The 35S:RUBY T‐DNA encodes the reporter *RUBY*, which converts tyrosine to vividly red betalain.

**Supplementary Figure S2.** **Comparison of plant height between wild‐type and transgenic *L. grandiflorum* L1**.
(A) Wild‐type and clonally propagated line 1 after 5 months of growth in the greenhouse.
(B) Plant height. WT: wild‐type *L. grandiflorum*, L1: transgenic line 1. The Asterisk indicates a significant difference from WT by Student's t‐test with *P < 0.05, n=6.

**Supplementary Figure S3.** **Mapping of T‐DNA insertions in transgenic *L. grandiflorum***.
(A) IGV snapshot of DNA‐seq reads mapping the 35S:RUBY plasmid. WT: wild‐type *L. grandiflorum*, L1, L2: two independent transgenic lines.
(B) IGV snapshot with coverage tracks of DNA‐seq reads mapping to the 35S:RUBY plasmid. WT: wild‐type *L. grandiflorum*, L1, L2: two independent transgenic lines.
(C) IGV snapshot with coverage tracks of DNA‐seq reads mapping to the pRiA4 plasmid. WT: wild‐type *L. grandiflorum*, L1, L2: two independent transgenic lines.

**Supplementary File S1.** Oligonucleotide sequences

**Supplementary File S2.** Identification of 35S:RUBY T‐DNA insertion site based on whole genome sequencing data.

**Data S1.** Detection of the presence of introduced transgenes using PCR

**Data S2.** Amplification of the T‐DNA insertion site using PCR

## Data Availability

The data that support the findings of this study are available from the corresponding author upon reasonable request
