# Supplementary material for: Establishment of an Agrobacterium‐mediated transformation system for the genetic engineering of Linum grandiflorum Desf
Source: Physiol Plant. 2025 Jan 20;177(1):e70059. doi: 10.1111/ppl.70059 (PMC11744441; doi:10.1111/ppl.70059)
Supplement: Supplementary file 2 — Supplementary Figure S1. Schematic representation of Agrobacterium rhizogenes and the Ri plasmid. Agrobacterium rhizogenes strain A4 is an agropine type strain that contains a root‐inducing (Ri) plasmid with two distinct T‐DNA fragments: TL‐DNA, which is involved in the biosynthesis of auxins, and TR‐DNA, which carries opine biosynthesis genes. Commonly used binary vectors, such as 35S:RUBY, are designed to carry foreign genes of interest and can be additionally introduced into the host plant genome. The 35S:RUBY T‐DNA encodes the reporter RUBY, which converts tyrosine to vividly red betalain. [file PPL-177-e70059-s006.pdf]

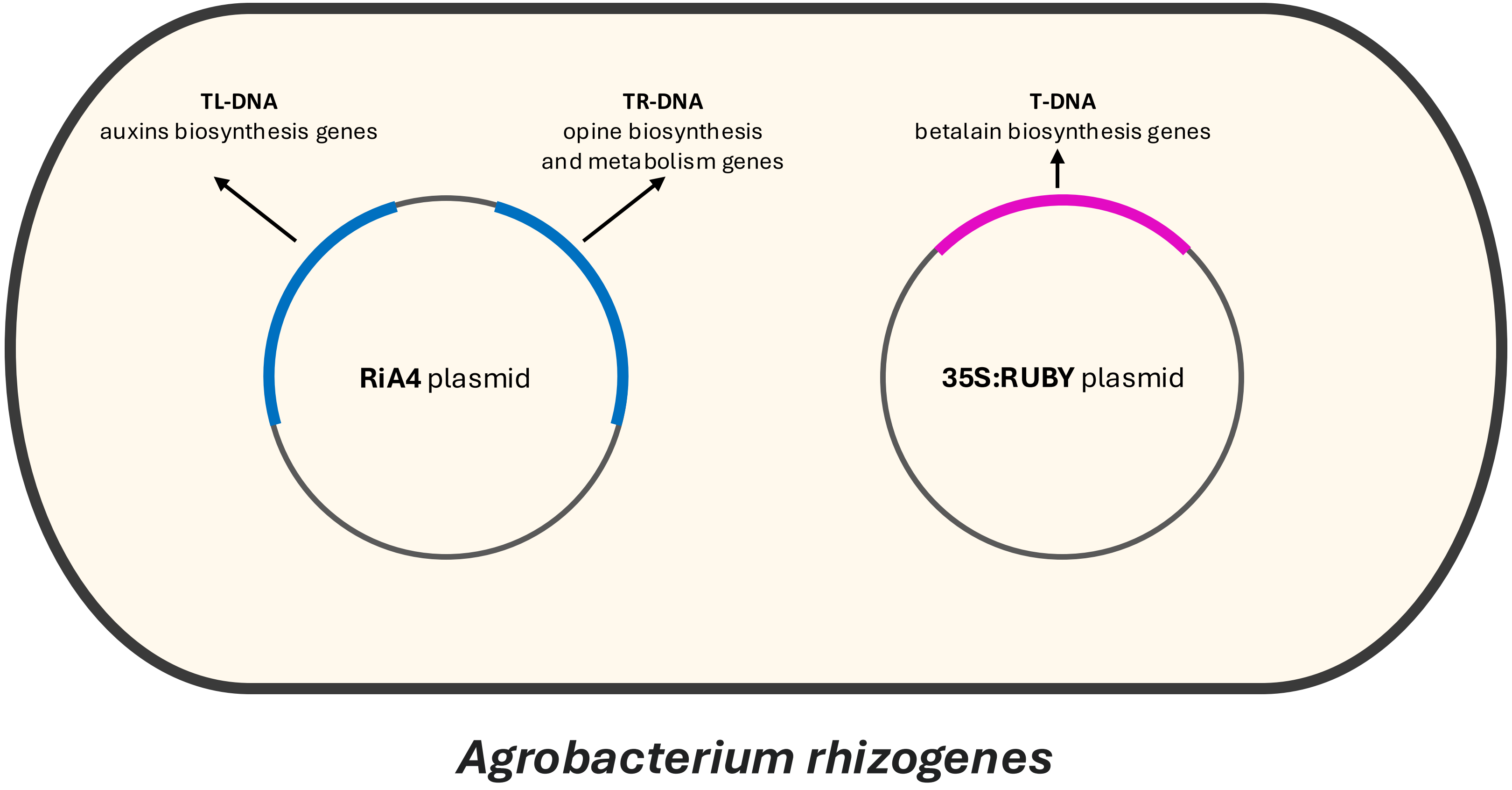

Supplementary figure S1. Schematic representation of *Agrobacterium rhizogenes* and the Ri plasmid.
Agrobacterium rhizogenes strain A4 is an agropine type strain that contains a root-inducing (Ri) plasmid with two distinct T-DNA fragments: TL-DNA, which is involved in the biosynthesis of auxins, and TR-DNA, which carries opine biosynthesis genes. Commonly used binary vectors, such as 35S:RUBY, are designed to carry foreign genes of interest and can be additionally introduced into the host plant genome. The 35S:RUBY T-DNA encodes the reporter *RUBY*, which converts tyrosine to vividly red betalain.
