# Supplementary material for: Establishment of an Agrobacterium‐mediated transformation system for the genetic engineering of Linum grandiflorum Desf
Source: Physiol Plant. 2025 Jan 20;177(1):e70059. doi: 10.1111/ppl.70059 (PMC11744441; doi:10.1111/ppl.70059)
Supplement: Supplementary file 3 — Supplementary Figure S2. Comparison of plant height between wild‐type and transgenic L. grandiflorum L1. (A) Wild‐type and clonally propagated line 1 after 5 months of growth in the greenhouse. (B) Plant height. WT: wild‐type L. grandiflorum, L1: transgenic line 1. The Asterisk indicates a significant difference from WT by Student's t‐test with *P < 0.05, n=6. [file PPL-177-e70059-s008.pdf]

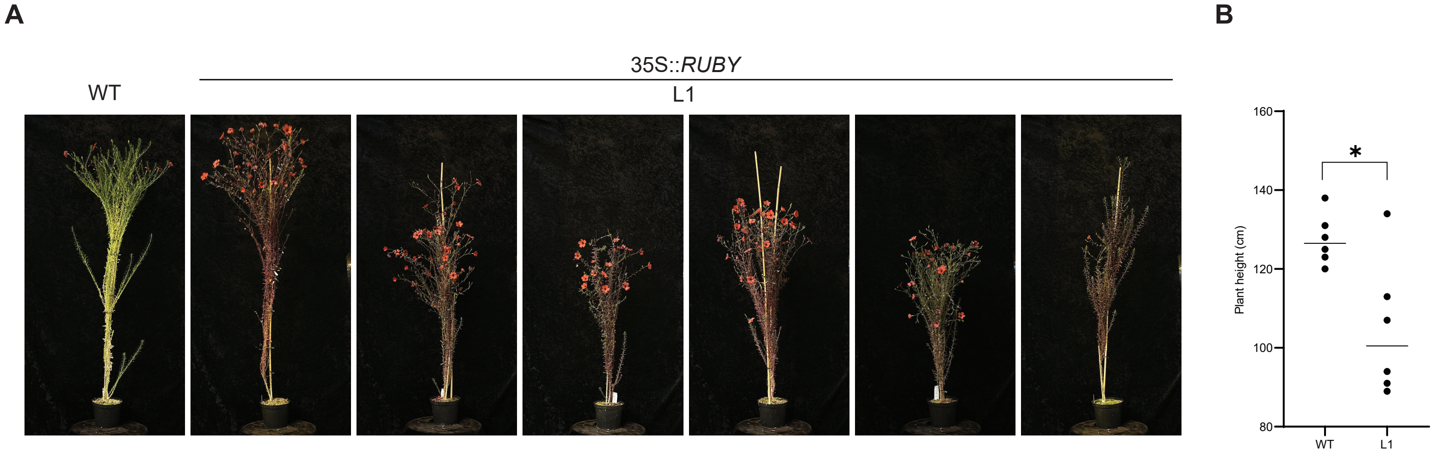

Supplementary Figure S2. Comparison of plant height between wild-type and transgenic *L. grandiflorum* L1.
(A) Wild-type and clonally propagated line 1 after 5 months of growth in the greenhouse.
(B) Plant height. WT: wild-type *L. grandiflorum*, L1: transgenic line 1. The Asterisk indicates a significant difference from WT by Student's t-test with *P < 0.05, n=6.
