# Supplementary material for: Establishment of an Agrobacterium‐mediated transformation system for the genetic engineering of Linum grandiflorum Desf
Source: Physiol Plant. 2025 Jan 20;177(1):e70059. doi: 10.1111/ppl.70059 (PMC11744441; doi:10.1111/ppl.70059)
Supplement: Supplementary file 4 — Supplementary Figure S3. Mapping of T‐DNA insertions in transgenic L. grandiflorum . (A) IGV snapshot of DNA‐seq reads mapping the 35S:RUBY plasmid. WT: wild‐type L. grandiflorum, L1, L2: two independent transgenic lines. (B) IGV snapshot with coverage tracks of DNA‐seq reads mapping to the 35S:RUBY plasmid. WT: wild‐type L. grandiflorum, L1, L2: two independent transgenic lines. (C) IGV snapshot with coverage tracks of DNA‐seq reads mapping to the pRiA4 plasmid. WT: wild‐type L. grandiflorum, L1, L2: two independent transgenic lines. [file PPL-177-e70059-s003.pdf]

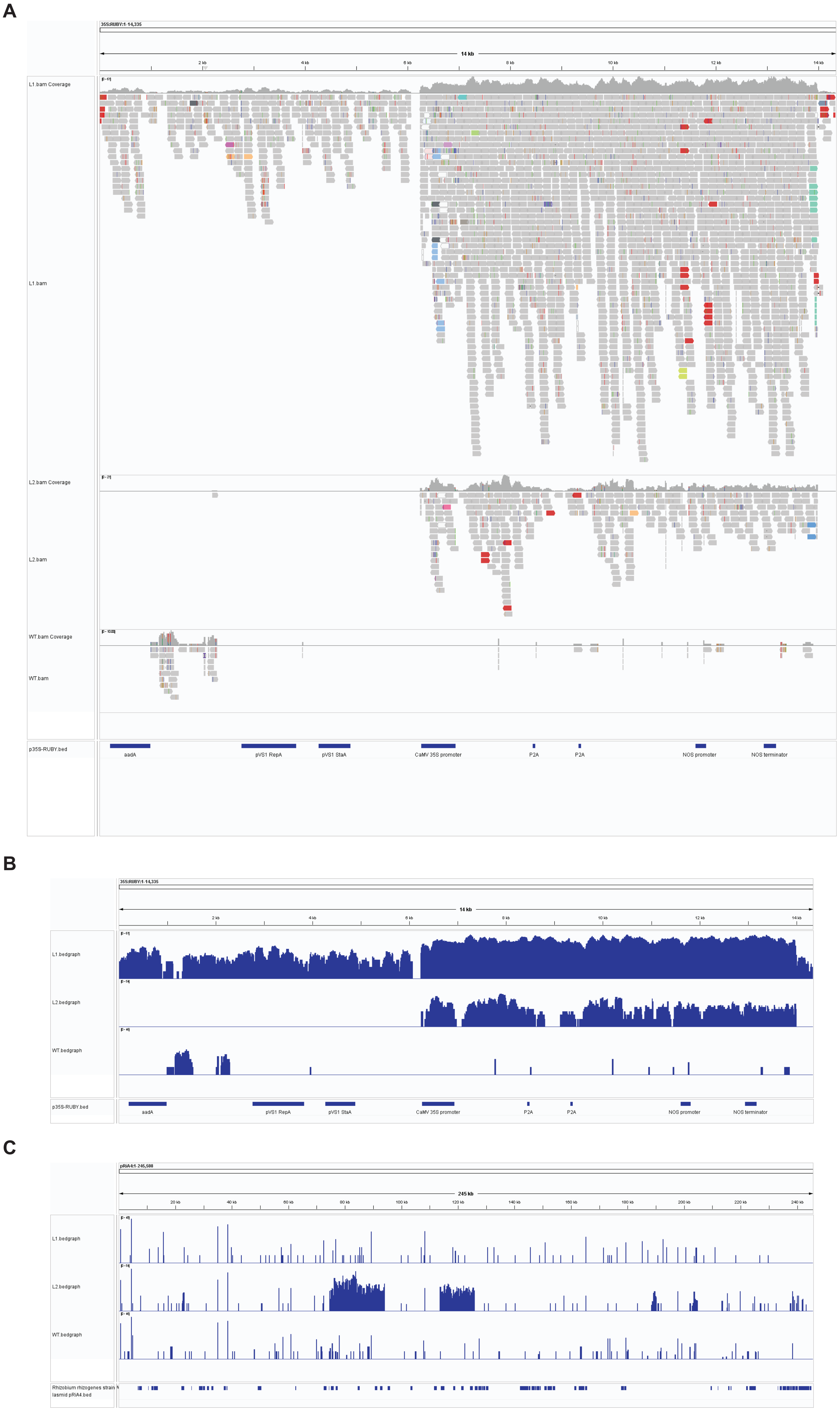

Supplementary Figure S3. Mapping of T-DNA insertions in transgenic *L. grandiflorum*.

(A) IGV snapshot of DNA-seq reads mapping the 35S:RUBY plasmid. WT: wild-type *L. grandiflorum*, L1, L2: two independent transgenic lines.

(B) IGV snapshot with coverage tracks of DNA-seq reads mapping to the 35S:RUBY plasmid. WT: wild-type *L. grandiflorum*, L1, L2: two independent transgenic lines.

(C) IGV snapshot with coverage tracks of DNA-seq reads mapping to the pRiA4 plasmid. WT: wild-type *L. grandiflorum*, L1, L2: two independent transgenic lines.
