# Supplementary material for: Establishment of an Agrobacterium‐mediated transformation system for the genetic engineering of Linum grandiflorum Desf
Source: Physiol Plant. 2025 Jan 20;177(1):e70059. doi: 10.1111/ppl.70059 (PMC11744441; doi:10.1111/ppl.70059)
Supplement: Supplementary file 5 — Supplementary File S1. Oligonucleotide sequences [file PPL-177-e70059-s004.docx]

**Supplementary file S1. Oligonucleotide Sequences**

Primers used for molecular analysis of transformed plants:

OKG15, hpt_fwd, ATTTGTGTACGCCCGACAGT

OKG16,  hpt_rev, CTCGGAGGGCGAAGAATCTC

OKG17, DODA_fwd, CCGTCGAGGATACACATCCG

OKG18, DODA_rev, TCCCAGGAGGAGTGGATCAG

OKG19, VirC1_fwd, CGCGATCCTGAGATTCCGTT

OKG20, VirC1_rev, GGAAAGAAAACGCCCTACGC

Primers used for amplification of 35S:RUBY T-DNA insertion site:

OKG53, L1-RUBY-L6487_fwd, AGAGGCCTACGGTATGTGGT

OKG54, L1-RUBY-L6487_rev_L1, TAGCTGGGCAATGGAATCCG

OKG55, L1-RUBY-L6487_rev_WT, TGCAGAGTGGTGGGGTTTTT
