# Supplementary material for: Establishment of an Agrobacterium‐mediated transformation system for the genetic engineering of Linum grandiflorum Desf
Source: Physiol Plant. 2025 Jan 20;177(1):e70059. doi: 10.1111/ppl.70059 (PMC11744441; doi:10.1111/ppl.70059)
Supplement: Supplementary file 6 — Supplementary File S2. Identification of 35S:RUBY T‐DNA insertion site based on whole genome sequencing data. [file PPL-177-e70059-s002.docx]

**Supplementary file S2.** Identification of 35S:RUBY T-DNA insertion site based on whole genome sequencing data.

WT locus-left:

GCTGTAATACAATGTCACGAAAGAGGCCTACGGTATGTGGTACNAAATCTAAACCGGTCCNACGGGTGGACNGATTAGCTGGTGGGGGTTCTCACGGGTGCATCATCTGGTAGGTACAATTCTAAAGGGAGTGTGGTGGGGGATCACAAGCAGAGAACACTAGAGAACAAACAGTAGCAAACAAAACAATCG

WT locus-right:

TAGCTCGAAAGATTCTGCAGCAGCAGTCACTGGAGAAAGAAAGAAAGAAGAGAGCTCTCTGACTGTCACCCTCGTGGCTGAGAAGAACCAATAGGTCAAAAAAACCCCACCACTCTGCAACTTGCAGAGCCTTTCACACCTCTCTCTGGTCAATAAAGCTTCATACTTTATCGTATTTTTCCCCACATTTTCCCTATGGTGTTTCATTATACGAATACTGAATTACTACTACTCTGTTAGATCTCTTCCCACGAG

L1 35S:RUBY T-DNA-right:

TCAAACACTGATAGTTTTGAGACTTTTCAACAAAGGGTAATATCGGGAAACCTCCTCGGATTCCATTGCCCAGCTATCTGTCACTTCATCAAAAGGACAGTAGAAAAGGAAGGTGGCACCTACAAATGCCATCATTGCGATAAAGGAAAGGCTATCGTTCAAGATGCCTCTGCCGACAGTGGTCCCAAAGATGGACCCCCACCCACGAGGAGCATCGTGGAAAAAGAAGACGTTCCAACCACGTCTTCAAAGCAAGTGGATTGATGTGAACATGGTGGAGCACGACACTCTCGTCTACT
