# Supplementary figures and images for: Establishment of an Agrobacterium‐mediated transformation system for the genetic engineering of Linum grandiflorum Desf
Source: Physiol Plant. 2025 Jan 20;177(1):e70059. doi: 10.1111/ppl.70059 (PMC11744441; doi:10.1111/ppl.70059)

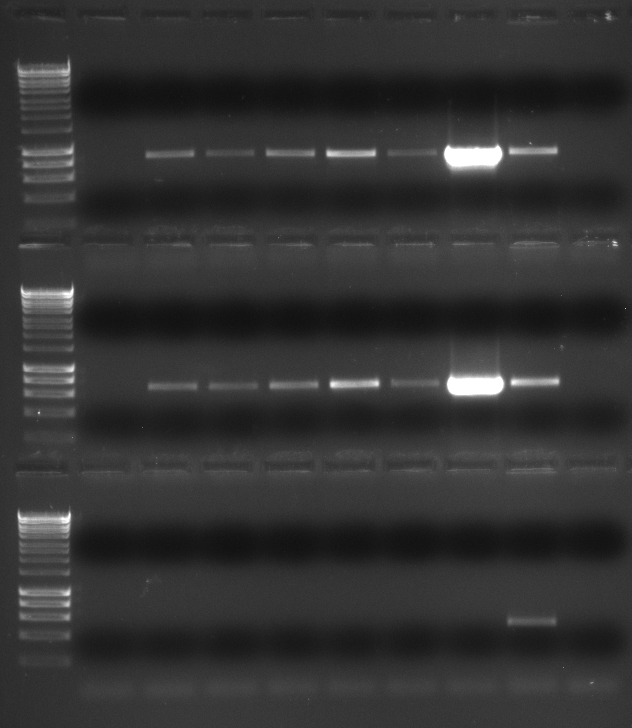

Supplement: Supplementary file 7 — Data S1. Detection of the presence of introduced transgenes using PCR [file PPL-177-e70059-s005.tif]

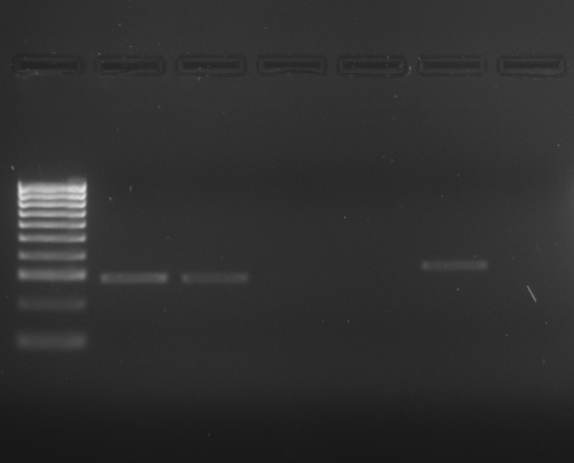

Supplement: Supplementary file 8 — Data S2. Amplification of the T‐DNA insertion site using PCR [file PPL-177-e70059-s007.tif]
